# Ecological and Health Risk Assessments of an Abandoned Gold Mine (Remance, Panama): Complex Scenarios Need a Combination of Indices

**DOI:** 10.3390/ijerph18179369

**Published:** 2021-09-05

**Authors:** Ana Cristina González-Valoys, José María Esbrí, Juan Antonio Campos, Jonatha Arrocha, Eva María García-Noguero, Tisla Monteza-Destro, Ernesto Martínez, Raimundo Jiménez-Ballesta, Eric Gutiérrez, Miguel Vargas-Lombardo, Efrén Garcia-Ordiales, Rosario García-Giménez, Francisco Jesús García-Navarro, Pablo Higueras

**Affiliations:** 1 Centro Experimental de Ingeniería, Technological University of Panama, Vía Tocumen, Panama City 0819-07289, Panama; jonatha.arrocha@utp.ac.pa; 2 Instituto de Geología Aplicada, Castilla-La Mancha University, EIMI Almadén, Plaza Manuel Meca 1, Almadén, 13400 Ciudad Real, Spain; JoseMaria.Esbri@uclm.es (J.M.E.); Eva.Garcia@uclm.es (E.M.G.-N.); Pablo.Higueras@uclm.es (P.H.); 3 Department of Geology & Geochemistry, Autonomous University of Madrid, University City of Cantoblanco, 28049 Madrid, Spain; raimundo.jimenez@uam.es (R.J.-B.); rosario.garcia@uam.es (R.G.-G.); 4 Escuela Técnica Superior de Ingenieros Agrónomos de Ciudad Real, Castilla-La Mancha University, Ronda de Calatrava 7, 13071 Ciudad Real, Spain; JuanAntonio.Campos@uclm.es (J.A.C.); FcoJesus.Garcia@uclm.es (F.J.G.-N.); 5 Departamento de Geotecnia, Facultad de Ingeniería Civil, Technological University of Panama, Ricardo J. Alfaro Avenue, Dr. Víctor Levi Sasso University Campus, Panama City 0819-07289, Panama; tisla.destro@utp.ac.pa (T.M.-D.); eric.gutierrez@utp.ac.pa (E.G.); 6 Dirección de Investigación, Vicerrectoría de Investigación, Postgrado y Extensión, Technological University of Panama, Ricardo J. Alfaro Avenue, Dr. Víctor Levi Sasso University Campus, Panamá City 0819-07289, Panama; ernesto.martinez@utp.ac.pa; 7 Facultad de Ingeniería de Sistemas Computacionales, Technological University of Panama, Ricardo J. Alfaro Avenue, Dr. Víctor Levi Sasso University Campus, Panamá City 0819-07289, Panama; miguel.vargas@utp.ac.pa; 8 SNI-SENACYT Sistema Nacional de Investigación-Secretaria Nacional de Ciencia, Tecnología e Innovación, Clayton, Ciudad del Saber Edif.205, Panama City 0816-02852, Panama; 9 Mining Exploration and Prospecting Department, University of Oviedo, Independencia Street, 13, 33004 Oviedo, Spain; garciaefren@uniovi.es

**Keywords:** potentially toxic elements (PTEs), pollution load index (PLI), potential ecological risk index (RI), dehydrogenase activity (DHA), human risks, Panama

## Abstract

The derelict Remance gold mine is a possible source of pollution with potentially toxic elements (PTEs). In the study area, diverse mine waste has been left behind and exposed to weather conditions, and poses risks for soil, plants and water bodies, and also for the health of local inhabitants. This study sought to perform an ecological and health risk assessment of derelict gold mining areas with incomplete remediation, including: (i) characterizing the geochemical distribution of PTEs; (ii) assessing ecological risk by estimating the pollution load index (PLI) and potential ecological risk index (RI); (iii) assessing soil health by dehydrogenase activity; and iv) establishing non-carcinogenic (HI) and carcinogenic risks (CR) for local inhabitants. Soil health seems to depend on not only PTE concentrations, but also on organic matter (OM). Both indexes (PLI and RI) ranged from high to extreme near mining and waste accumulation sites. As indicated by both the HI and CR results, the mining area poses a health risk for local inhabitants and particularly for children. For this reason, it will be necessary to set up environmental management programs in the areas that are most affected (tailings and surrounding areas) and accordingly establish the best remediation strategies to minimize risks for the local population.

## 1. Introduction

Mining activities can potentially pollute the environment, especially when tailing materials are left exposed to weather conditions, which favor the release and dispersion of pollutants to the surrounding soil, plants, water bodies and humans [1,2,3,4]. The dispersion of potentially toxic elements (PTEs) can be primarily assessed by edaphological characterization and geochemical quantification of pollutants. However, assessing the possible impact on biota requires that certain indices be determined, such as the pollution load index (PLI) to evaluate the degree of contamination; the potential ecological risk index (RI) to evaluate the ecological risk that mine materials can pose [5]. Soil health is the capacity of the soil to function as a vital living ecosystem to sustain plants, animals, and humans [6]. Indicators of soil health provide information about how the soil is functioning with respect to a particular management goal or ecological role [7]. A specific soil function may involve several processes, and each process may be associated with a combination of soil chemical, physical, and biological properties [7]. In this sense, dehydrogenase activity (DHA) is a biological indicator that, added to the rest of the physical and chemical properties measured in this study, allows us to evaluate the health of the soil. Dehydrogenase activity (DHA), as an indicator of “soil health” [8], acts as a monitor of microbiological redox systems and is considered an adequate measure of microbial oxidative activities in soil [9]. DHA also plays a significant role in the biological oxidation of organic matter (OM) by transferring hydrogen from organic substrates to inorganic acceptors [10] and is affected by several factors such as soil moisture, oxidation reduction potential (ORP), reactivity (pH), OM, soil profile depth and concentrations of PTEs [4,9,11]. Thus, it is important to measure these factors and to analyze their relation to DHA.

PTEs in mining areas can be of natural origin because they are components of rocks and ore minerals, whether the area has been exploited or not [12]. Mining activity can promote the distribution of these PTEs (Cu, Zn, As, Sb, Ba, Hg) on the surface and increase their concentrations as a result of mineral weathering, which would increase their potential toxicity [13]. It is necessary to recognize that some PTEs released through mining activity (e.g., Cu, Zn) are also essential elements for life, but are toxic in excess, while even low concentrations of other non-essential (e.g., Hg, Pb) elements are toxic for the environment [14,15]. Some of the pollutants found at high concentrations in the Remance area (As, Hg, Zn, Cu, Ba, Sb and cyanide) are on the priority list of the Agency for Toxic Substances and Disease Registry because these substances can potentially affect human health depending on their toxicity, frequency and exposure at polluted sites [16]. All of these pollutants can cause various health problems: arsenic (As) can cause skin, liver and lung cancers; mercury (Hg) produces neurological damage; zinc (Zn) can weaken the immune system; copper (Cu) can cause abnormalities to the nervous system; barium (Ba) can favor muscle paralysis; antimony can harm the respiratory system [17]; and cyanide can cause headaches and enlarge the thyroid gland, even at low concentrations [18]. Other possible effects of PTEs include carcinogenic and non-carcinogenic ones [1,19].

The objective of this study was to characterize the geochemical distribution of these pollutants in soil and fluvial sediment (including active channel stream sediments and terrace sediments). An ecological and health risk assessment was performed by estimating the PLI and RI indices. Besides DHA, an edaphological characterization was used to provide soil health information. The ultimate objective was to gain complete insights to assess the local risks for human health in this area.

## 2. Materials and Methods

### 2.1. Study Area

The Remance gold mine is located in the province of Veraguas in central Panama. The weather in this area is AMI type according to the Köppen-Geiger classification: a humid tropical climate, with the influence of monsoons and annual rainfall >2250 mm, concentrated (60%) in the four wettest months (August–November). Dry months (January–March) have rainfall rates below 60 mm, and the average temperature of the coolest month is >18 °C [20,21]. The topography in the area is quite irregular, with an altitudinal range between 150 and 266 m.a.s.l. that corresponds to the Veneno stream mouth and the maximum height of hills Principal and Tullido, respectively. The stream is called the Veneno (Poison) because the waters from the first tailings facility used by the Minera Remance company are discharged into it along with the water that flows from the pithead. Plant cover corresponds mostly to bushes, with areas delimited for small-scale agriculture, with rotary burning for crop cultivation and cattle farming [22].

**Figure 1:**
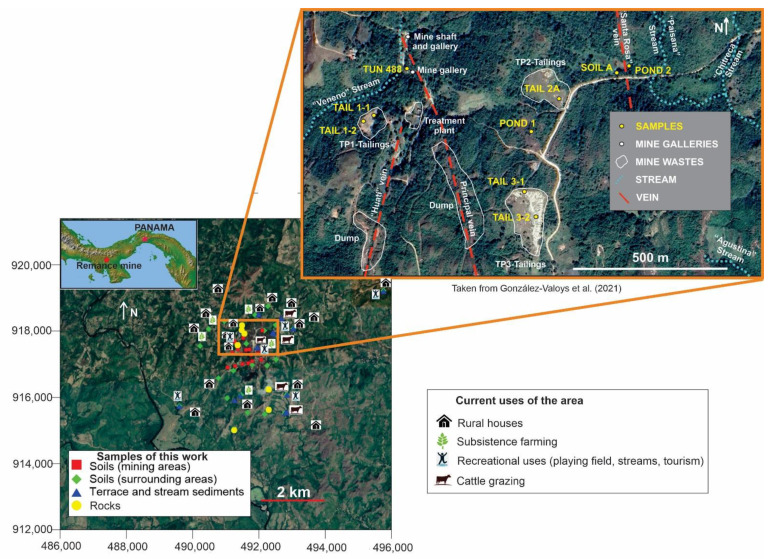
Location map of the samples taken at the Remance gold mine site and use of the area.

The exploited mineralization corresponds to epithermal gold hosted in pyroclastic rocks, which include several veins distributed in an area covering approximately 10 km^2^ [23] (Figure 1). Mining exploitation has occurred there intermittently since 1800, but we were unable to find details on its extraction processes. The Veraguas Mining Company produced 15,500 tons with 10.5 g of gold extracted per ton from the Remance mine during the 1800s; the Panama Corporation produced another 70,000 tons with 12 g of gold extracted per ton between the years 1923 and 1932 [23]. The last company to operate the site using the cyanidation process [22] was Minera Remance S.A., between 1989 and 1999 [23]; for this period, it reported cumulative production of 53,480 ounces of gold [24]. During this period, many complaints were filed regarding pollution of local water bodies and the Santa María River [22,24]. Presently (June 2021), there is a plan to reinitiate mining activity, which has led to local discord [25].

A drainage network runs through the area, and includes the Veneno stream, the Chitreca stream and the La Máquina stream on the slopes of Hill Tullido, excavated to exploit a quartz stockwork [23]. Possible sources of pollution in the area include mine excavations, which correspond mostly to surface trenches following mineralized veins. Au occurs as small inclusions within pyrite and marcasite, as well as free gold, disseminated within quartz, together with other accessory minerals in small quantities such as chalcopyrite, sphalerite, galena and arsenopyrite. Ag and As are found with Au, along with anomalous amounts of Sb and locally Hg [23]. Other polluting sources are represented by a mine gallery with its continuous water flow, a number of scattered dumps as a result of excavating trenches that evidently contain some proportions of exploited ore minerals and three tailing dams used to concentrate gold ore by means of cyanidation and containing high concentrations of PTEs (Cu, Zn, As, Sb, Ba, Hg, T-CN) according to studies carried out by González-Valoys et al. (2021) [26]. They are all currently exposed to local tropical climate conditions, such as rain (more than 2200 mm/year) and high temperatures, which favor chemical changes in ore and gangue minerals [4], and wind, which favors the aerial dispersion of pollutants [27]. The area is inhabited by peasants, who engage in subsistence activities such as agriculture and livestock farming [22,28]. Hence, the Remance area can be considered a rural, agricultural and recreational site (Tailing 2 is used as a soccer field by inhabitants, and people visit the area to see the old mine; in rural areas, streams are often used for recreational purposes and for water intake for livestock).

### 2.2. Sampling

Samples were collected between May–June 2019 (wet season) and January 2020 (dry season), and included the following the types:

- 43 soil samples, including 19 samples collected in the mining area around the tailings and excavations of the mine’s veins and tunnels, and 24 samples collected within the perimeter of the mine and its surroundings;
- 39 sediment samples, including 19 fluvial active channel stream sediments and 20 fluvial terrace sediment samples taken from the banks of water bodies;
- 7 rock samples collected from outcrops to acquire data about the general lithology in the area.

In order to make a comparison to the regional base level, a background sample for each material type (soil, terrace sediment and stream sediment) was taken approximately 4 km from the mining area, near the town of El Naranjal. Figure 1 shows the locations of samples, mine structures and waste accumulation areas [26]. Table S1 displays the sample codes and typology, along with geographic coordinates.

Soil samples were taken using a PVC tube at a depth of 0–30 cm from the potentially most affected zone. Sediment samples were collected with plastic shovels from the top 5–10 cm. All of the samples weighed about 3 kg each and were stored in plastic bags at room temperature prior to sample preparation.

### 2.3. Sample Preparation and Analysis

All of the samples were dried at room temperature in the laboratory, disaggregated and passed through a 2 mm sieve. Afterward, some representative aliquots were extracted according to different analytical determinations. The subsamples taken to determine DHA were transported in hermetically sealed bags and were cold-stored. An aliquot of the samples taken at <2 mm (50 g) was crushed in an agate mortar to a <100 µm grain size to determine the concentrations of PTEs (Cu, Zn, As, Sb, Ba, Hg) and for mineralogy identification purposes. Another aliquot was used to determine certain edaphic parameters: pH, electrical conductivity (EC), ORP. The methodology to perform these analyses included a 1:5 suspension (*w*/*v*) (ASTM D 4972) [29], and determination was performed using a multi-parameter benchtop Orion Versa Star Pro device. Other established edaphological parameters included OM, quantified by weight loss at 455 °C (ASTM D 2974) [29], and cationic exchange capacity (CEC), established by the potentiometer method [30]. Color when wet was measured by Munsell soil charts. The soil texture classification was determined by the Bouyoucos method described by Porta [31].

The concentrations of Cu, Zn, As, Sb and Ba were analyzed by energy dispersive X-ray fluorescence spectroscopy (ED-XRF) with Epsilon1 equipment (Panalytical brand) [4]. Total Hg (THg) was determined by Zeeman atomic absorption spectroscopy with high-frequency modulation of light polarization (ZAAS-HFM) using commercial equipment Lumex RA-915 M with a pyrolytic attachment (PYRO-915+) [32]. Certified reference material was employed to check both precision and accuracy: NIST 2710A (Montana soil), with recovery percentages between 95–105%. The analysis of total cyanide, cyanide complexes and easily leachable cyanide was performed by the ALS Global laboratory using the cyanide (CN) complexes according to Standard UNE-EN ISO 14403-2 [33].

DHA was determined by the triphenyltetrazolium chloride (TTC) method [34,35,36] on samples selected after considering variabilities in As and Cu to assess the influence of the concentrations of these PTEs on this enzymatic activity. Dehydrogenase converts 2,3,5-TTC into triphenyl formazan (TPF) [36]. The homogenized soil samples (1.5 g) were placed inside test tubes (15 × 120 mm) and were mixed with 1.5 mL of deionized water, 0.015 g of CaCO_3_ and 0.250 mL of TTC (3% *v*/*w*) to be vortexed (2 min) and incubated (Memmert In 30) at 37 °C for 24 h. Next, samples were vortexed (4 min) with methanol used as the extractor agent (5 mL). Tubes were centrifuged (Ortoalresa, Unicen 21) at 4000 rpm for 10 min, and the supernatant was obtained and analyzed in a UV visible spectrophotometer (Biochrom, Libra S60) at 485 nm. The results were expressed as µg TPF d^−1^ g^−1^.

Mineralogy was analyzed by X-ray powder diffraction (XRD) using a PAN analytical X-Pert PRO X-ray diffractometer fitted with a Cu anode. The operating conditions were 40 mA, 45 kV, 0.5° divergence slit and 0.5 mm reception slits. Samples were scanned with a step size of 0.0167° (2θ) and 150 ms per step. Characterization of samples was performed by the powder method between 5 and 55° (2θ). The Match v.3 and Fullprof software for the Rietveld analysis were used for the quantification [37,38,39]. The Crystallography Open Database (COD) reference patterns were utilized to identify mineral phases.

### 2.4. Methods

#### 2.4.1. Pollution Index and Pollution Load Index

Soil quality can be estimated by various indices [40]. A site polluted by a certain element can be assessed by the pollution index (PI) (Equation (1)), and the same, but with more than one element, can be determined by the pollution load index (PLI) [5,41] using Equation (2).

PI = C _soil_/C _background_ (1)

PLI = (PI_1_ × PI_2_ × … × PI_n_)^1/n^ (2)

where C_soil_ and C_background_ are the concentrations of the particular PTE in the soil and background samples, respectively (mg kg^−1^). The PLI is the pollution load index of several elements, and the PI is the single pollution index for a certain element. Table 1 presents the evaluation criteria for the PLI [5].

#### 2.4.2. Toxicity Response Coefficient and the Potential Ecological Risk Index

Hakanson [42] defined RI as an index that combines environmental effects and element toxicity with the aim of considering the general ecological migration and transformation trends of these elements in soils and sediments. Pan et al. (2019) [5] defined the toxicity response coefficient (Er) as the single potential ecological risk for a certain element (Equation (3)), which is used to obtain RI (Equation (4)):

Er^i^ = PI × Tr^i^ (3)

RI = Er_1_ + Er_2_ + … + Er_n_ (4)

where Er^i^ is the single RI, PI is the single pollution index for a certain element and Tr^i^ is the toxicity response coefficient of element i. The Er calculation is based on Hakanson’s element toxicity response coefficient standards [42], which can be given as follows: Hg = 40, As = 10, Cu = 5, Zn = 1 and Sb = 7 [43]. Table 1 summarizes the evaluation thresholds for these indices based on the respective proposers.

#### 2.4.3. Human Health Risk Assessment

Some PTEs released from mining waste and incorporated into different environmental compartments can have an immediate effect on human health. The assessment was performed by using the average daily doses (ADD) through three pathways: accidental soil ingestion (ADD soil ingestion); skin contact (ADD dermal contact) [19,44,45,46]; and soil dust inhalation (ADD inhalation) [1,47]. Additionally, both the HI and CR risks were assessed in different scenarios: residential, agricultural and recreational [19,48].

ADDs were calculated as mg kg^−1^ day^−1^ as follows (Equations (5)–(7)):

ADD soil ingestion = (C_soil_ × CF × IR_ing_ × FI × EF × ED × RBA)/(BW × AT) (5)

ADD dermal contact = (C_soil_ × CF × AF × ABS_d_ × EF × ED × EV × SA)/(BW × AT) (6)

ADD inhalation = (C_soil_ × IR_air_ × EF × ED)/(BW × AT × PEF) (7)

where C_soil_ is the concentration of PTEs in soil in mg kg^−1^; CF is the conversion factor (1 × 10^−6^ kg mg^−1^); BW is body weight (70 kg in an adult, 15 kg in a child); IR_ing_ is the ingestion rate in soil; FI is fraction ingested; EF is exposure frequency; ED is exposure duration; RBA is the relative bioavailability factor (unitless); AT is the averaging time; AF is the adherence factor; ABS_d_ is the dermal absorption factor (unitless); EV is event frequency; SA is the skin surface area; IR_air_ is the inhalation rate; and PEF is the soil-to-air particulate emission factor (m^3^ kg^−1^). All of these parameters are presented in Table 2 for the three exposure pathways in three scenarios. The constants for the PTEs used in the study appear in Table 3.

##### Non-Carcinogenic Risk

The HI risk was calculated with the hazard quotient (HQ) as follows (Equation (8)):

HQ = ADD/RfD (8)

where ADD is the average daily dose for soil ingestion, dermal contact or inhalation as mg kg^−1^ day^−1^, and RfD is the reference dose for oral, dermal or inhalation as mg kg^−1^ day^−1^. Table 3 presents the RfD values for the three exposure pathways: RfDo (oral reference doses), RfDd (dermal reference doses) and RfDinh (inhalation reference doses). RfDd was calculated according to Wcislo et al. (2016) [49] and Gruszecka-Kosowska et al. (2020) [19].

The total HI risk of PTEs was determined by the hazard index (HIt) [48], as Equation (9) shows:

HIt = HQ1 + HQ2 + … + HQn(9)

where HQs are the hazard quotient values for the 1–n PTEs herein investigated.

##### Carcinogenic Risk

CR risks were calculated as follows (Equation (10)):

CR = ADD × SF (10)

where ADD is the average daily dose for soil ingestion, dermal contact or inhalation as mg kg^−1^ day^−1^ and SF is the slope factor for oral, dermal or inhalation over a lifetime for a particular PTE that plays a key role in daily toxin intake and results in an increased risk of an individual developing cancer [1,5]. Table 3 presents the SF values for the three exposure pathways. SFd was calculated according to Wcislo et al. (2016) [49] and Gruszecka-Kosowska et al. (2020) [19].

The total CR risks of PTEs were determined according to the CRt values [48] as follows (Equation (11)):

CRt = CR1 + CR2 + … + CRn (11)

where CRt are the CR risk values for the 1–n PTEs investigated in this study.

The total risk for both the HI and CR risks was calculated by the sum of the risks for the different exposure pathways: (Equation (12)):

Risk (total) = Risk (ingestion) + Risk (dermal) + Risk (inhalation) (12)

#### 2.4.4. Statistical Analyses

Microsoft Excel spreadsheets were used to manage the results. Minitab 15 was employed to analyze the statistical parameters of the analytical results. A multivariate analysis was performed by applying Ward’s linkage to obtain significant dendrograms. A factor analysis and a principal component analysis (PCA) were applied to search for the influence of factors, or group of factors, using “Varimax” orthogonal rotation.

The distribution maps of PTEs, PLI, RI, HI and CR were generated with the Surfer 9 software, licensed by the UCLM, using the option “inverse distance to a power (2)” to generate the corresponding distribution maps.

## 3. Results

### 3.1. Total Contents

The average concentrations of the PTEs from the different material types in the sampled Remance gold mine areas are presented in Table 4, while all of the obtained results appear in Table S2. pH ranged between 3.9 and 5.9. The most acidic value was found in pithead sediments and the most neutral one in the mining work areas. EC ranged between 0.03 to 0.52 dS m^−1^, with the lowest value in the mining area and the highest value in pithead sediments. OM varied between 0.8% and 12.9%, with the smallest amount in tailings and the largest amount in pithead sediments.

The concentrations of Cu varied between 10.9 and 403.0 mg kg^−1^, and those of As from 41.0 to 5030.0 mg kg^−1^, with the lowest concentration in cyanidation ponds and the highest in pithead sediments. Cyanidation ponds are the final part of the process, and then join the streams [22]; these have been exposed to rain currents for more than 20 years. Pithead sediments are sediments that come out of the galleries of the mine, located in the “principal” vein, where underground water flows; this water current is then incorporated into a “Veneno” stream. Zn concentrations varied between 27.0 and 153.0 mg kg^−1^, with the lowest in mine tailings and the highest in pithead sediments. Sb concentrations ranged from 1.4 to 23.2 mg kg^−1^, and those of Ba between 55.4 and 398.7 mg kg^−1^, with the lowest in cyanidation ponds and the highest in stream sediments for both elements. Hg concentrations ranged from 0.06 to 1.37 mg kg^−1^, with the lowest in soils from the surrounding areas and the highest in mine tailings. The last tailings were from mining operations in 1999. Mercury is one of the elements present in mineralization in small amounts [23], which is why it can be present in tailings after mining.

The order of the average concentration of PTEs was: Ba > As > Cu > Zn > Sb > Hg in stream sediments and terrace sediments, Ba > Cu > As > Zn > Sb > Hg in the soils from the mining area and Ba > Cu > Zn > As > Sb > Hg in the soils from the surrounding area.

The average PTE concentrations for rocks from outcrops, as determined by ED-XRF (ST2), were: Cu (40.3 mg kg^−1^), Zn (22.8 mg kg^−1^), As (122.7 mg kg^−1^), Sb (25.7 mg kg^−1^), Ba (239.7 mg kg^−1^) and T(Hg) (0.17 mg kg^−1^).

### 3.2. Spatial Variability

**Figure 2:**
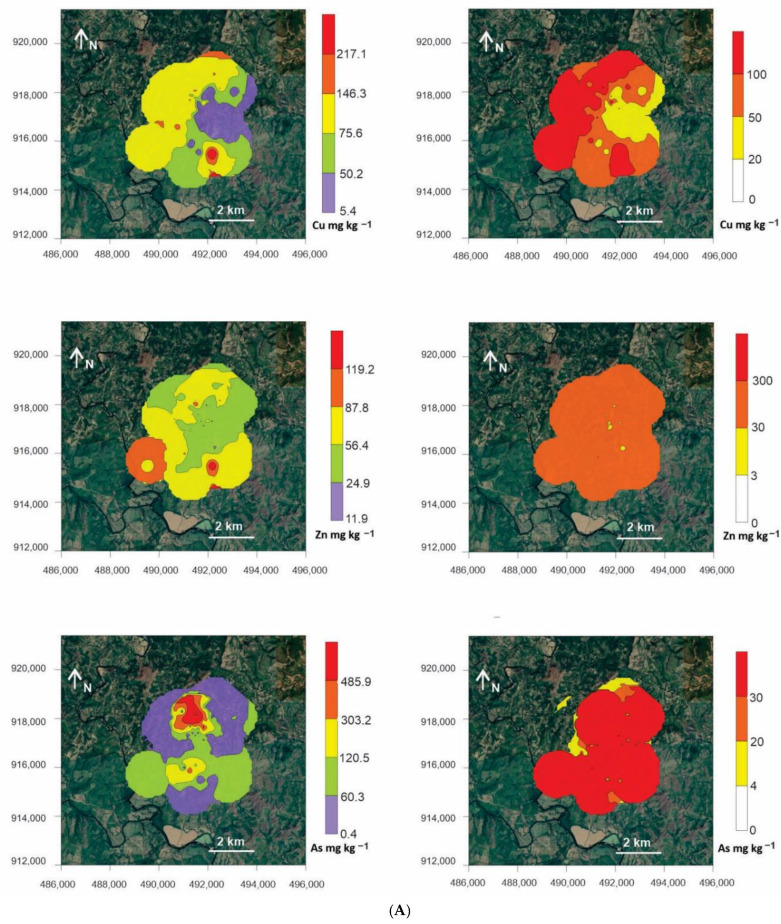

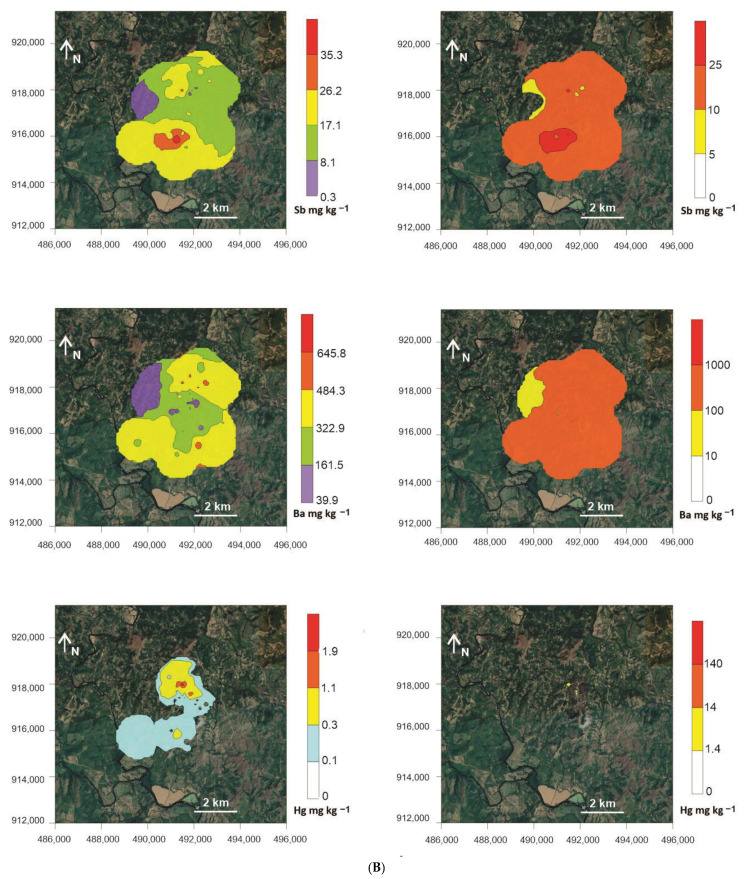
(**A**) Distribution maps for Cu, Zn, and As. Left: according to the statistical figures; right: according to the soil guideline values listed in Table 5 (yellow: agricultural limit; orange: residential limit; red: industrial limit). Distribution maps of PTEs generated using the concentration values for soils (surrounding areas), soils (mining areas), stream sediments, terrace sediments, tailings, cyanidation ponds and pithead sediments. (**B**) Distribution maps for Sb, Ba and Hg. Left: according to the statistical figures; right: according to the soil guideline values listed in Table 5 (yellow: agricultural limit; orange: residential limit; red: industrial limit). Distribution maps of PTEs generated using the concentration values for soils (surrounding areas), soils (mining areas), stream sediments, terrace sediments, tailings, cyanidation ponds and pithead sediments.

Isoconcentration maps were generated to analyze the geographical distribution of the analytical results. One set of maps indicates the concentration thresholds (minimum value, average − 1σ, average, average + 1σ, average + 2σ), while another set displays the results according to the guideline values indicated in Table 5 for agricultural, residential and industrial uses (Zn, As, Ba and Hg according to the Panama Standard; Cu and Sb according to the Costa Rica Standard). The PTE concentrations were high over almost the entire perimeter of the study area, and surpassed the Cu and As guideline values, even for industrial-use levels, while Zn, Sb and Ba exceeded the values set out in the standard for residential use. The exception was Hg, which exceeded the value for agricultural use only in tailings (Figure 2A,B).

### 3.3. Mineralogical Analysis

Table 6 presents the estimated percent abundance of the identified minerals for the selected samples from soils, terrace sediments and stream sediments. The diffractograms of studied samples are compiled in Figure S1. The main mineral phases identified in soils were quartz (89%), kaolinite (10%) and illite (1%), while terrace sediments were composed of quartz (84%), kaolinite (11%), feldspar (3%), illite (1%) and chlorite (1%). Stream sediments comprised quartz (84%), kaolinite (11%), and chlorite (5%). The found mineral phases were similar in soils and sediments (terrace sediments and stream sediments) and the main phases were quartz and kaolinite. The presence of PTEs could be associated with clay fractions, such as kaolinite and illite [53], due to no characteristic minerals of these PTEs being detected in the XRD analysis. Consequently, the most plausible explanation is the absorption/adsorption of the PTEs by clays, as they were the minerals with the highest representation.

### 3.4. DHA and Correlations with Edaphic Parameters and PTEs

The DHA values were higher in soils than in terrace sediments and were lower in stream sediments (63.27 > 37.47 > 24.79 µg TPF g^−1^ d^−1^), contrary to what was reported in soils and sediments in Morro do Ouro, the largest industrial gold mine in Brazil [54]. The DHA values of the present work were lower than those measured in other contaminated soils in the Ventanas Cu smelter, situated in the Puchuncaví Valley of Central Chile (110 mg TPF g^−1^ d^−1^) [55], in Almadenejos located in the mining district of Almadén, Spain (484 mg TPF g^−1^ d^−1^) [36], and in restored soils from mine sites (140–580 mg TPF g^−1^ d^−1^) reported by Mukhopadhyay and Maiti (2010) [56]. To seek correlations between pollution of the area and its biological activity, a statistical analysis was performed. Table 7 presents the average values of the DHA results, the physicochemical parameters, and the concentrations of PTEs, total cyanide (T-CN), complex cyanide (C-CN) and easily releasable cyanide (E-CN) for soils, terrace sediments and stream sediments. The same trend was found in the OM (5.3 > 4.2 > 3.4%) and CEC (10.61 > 9.74 > 9.08 cmol kg^–1^) data, while As showed higher concentrations in stream sediments and terrace sediments and lower concentrations in soils (188.9 > 146.5 > 55.7 mg kg^−1^). Some elements (Cu and Zn) presented higher concentrations in soils than in sediments, while total cyanide presented a more complex fractionation.

### 3.5. Multi-Elemental Analysis

**Figure 3:**
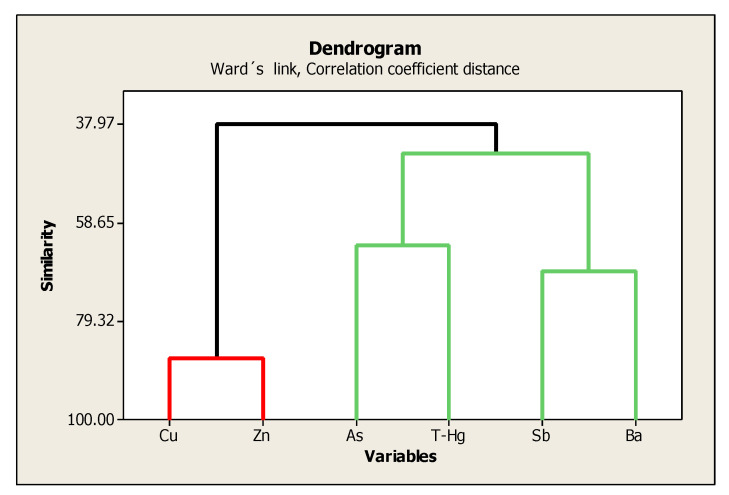
Dendrogram of the relations between the concentrations of PTEs in the study. Dendrogram generated with the data from soils, terrace sediments and stream sediments.

**Figure 4:**
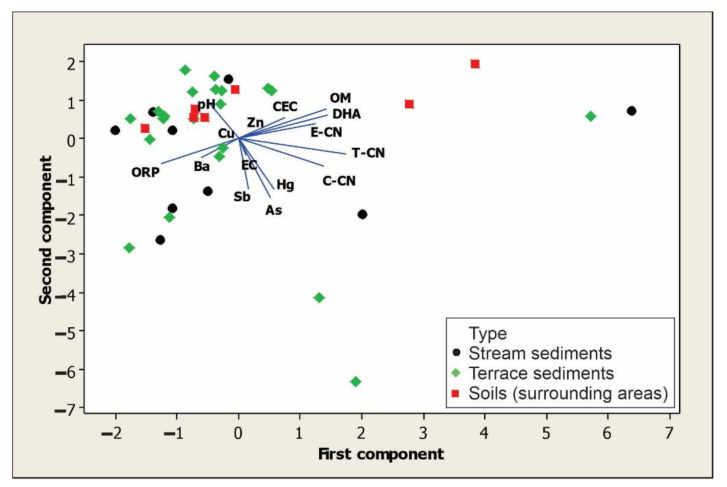
DHA and relationship with edaphic parameters, PTEs and cyanide. Generated with the data from soils, terrace sediments and stream sediments.

According to the mineralogical analysis, the samples were very similar (soils, terraces sediments and stream sediments), which is why we grouped them all together to perform the dendrogram and PCA. In the dendrogram obtained from the cluster analysis (Figure 3), Cu appears to be associated with Zn in one group, while As, Hg, Sb and Ba appear in another group. To determine the strength of the relationships between DHA and edaphic parameters, multivariate principal component analysis (PCA) was used (Figure 4, Table 8). Although the relationships were not so strong, the most significant in the first main component (PC1) and positively related to DHA (0.384) were T-CN (0.464), OM (0.379), C-CN (0.369), E-CN (0.333) and CEC (0.202), and negatively related to ORP (−0.334) and Ba (−0.160); while in the second principal component (PC2), As (−0.489), Sb (−0.424) and Hg (−0.423) were negatively related.

### 3.6. Pollution Index (PI) and Pollution Load Index (PLI)

**Figure 5:**
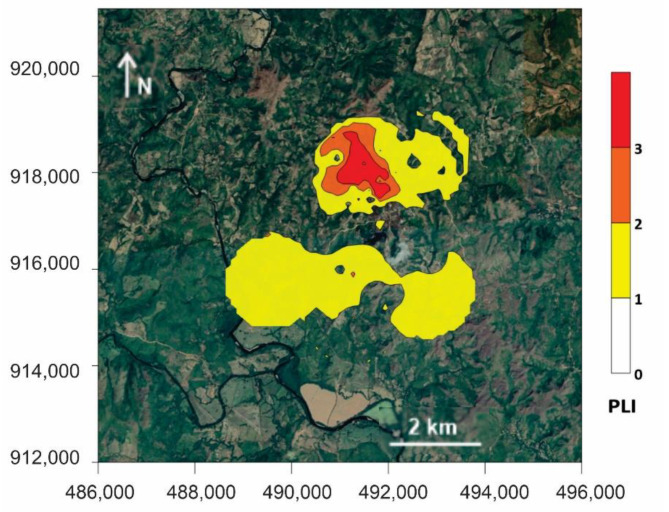
Distribution map of PLI for Cu, Zn, As, Sb, Ba and Hg. Distribution maps of PLI generated using the concentration values for soils (surrounding areas), soils (mining areas), stream sediments, terrace sediments, tailings, cyanidation ponds and pithead sediments.

Table 9 presents the PI and PLI values, and Figure 5 provides the PLI map. According to the evaluation criteria for PI and PLI presented in Table 1, the PI indicated serious pollution (PI > 3) in the different studied areas. Within these, the tailings and the pithead that showed the highest PI values due to the materials of both areas were the main sources of PTEs in the nearby environment.

The PLI followed this order: pithead sediments > tailings > terrace sediments > stream sediments = cyanidation ponds > soils from the mining area and soils (from the mining and surrounding areas). The PLI map shows an area with moderate to considerable damage corresponding to and around the tailings and pithead area, followed by slight damage to the surrounding area and south of the mining concession.

### 3.7. Toxicity Response Coefficient (Er) and Potential Ecological Risk Index (RI)

**Figure 6:**
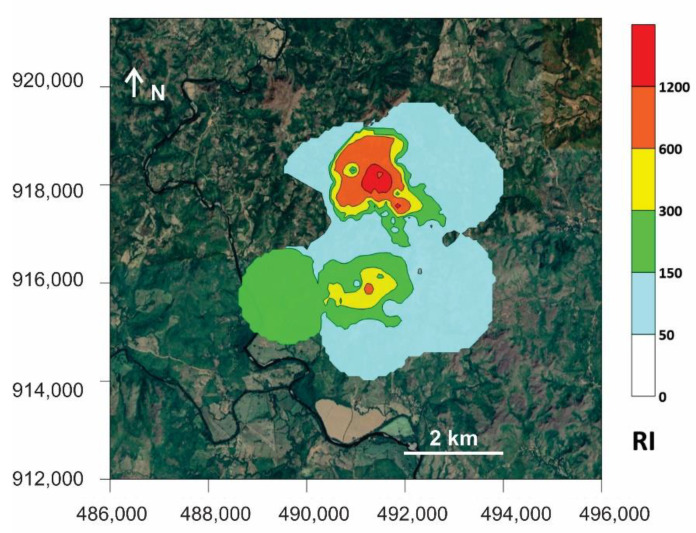
Geographical distribution of RI for Cu, Zn, As, Sb and Hg. Distribution maps of RI generated using the concentration values for soils (surrounding areas), soils (mining areas), stream sediments, terrace sediments, tailings, cyanidation ponds and pithead sediments.

The values calculated for Er and RI of Cu, Zn, As, Sb and Hg are shown in Table 10, while Figure 6 presents the geographical distribution for RI. According to the evaluation criteria presented in Table 1 for Er and the toxicity according to each element, the Er values suggests that tailings have a damaging role in the area, as well as the pithead; thus the terrace sediments and stream sediments have been significantly impacted and represent important sources of risk due to their content of As, Hg and Sb; in the case of soils from the mining area, Hg is the only major concern.

The average RI values and their distribution indicate a gradation in risk as follows: pithead sediments (extreme risk); tailings and terrace sediments (serious risk); stream sediments (high risk); cyanidation ponds, soils from the mining area and soils from the surrounding areas (mild risk). One remarkable finding indicated that the areas at higher risk coincided with those with higher PLI values for As, Sb, Ba and Hg (Figure 5), with an extensive area representing a serious to extreme RI that also corresponded to the area near tailings and pithead sediments.

### 3.8. Human Health Risk Assessment

#### 3.8.1. Non-carcinogenic Risk (HI)

**Figure 7:**
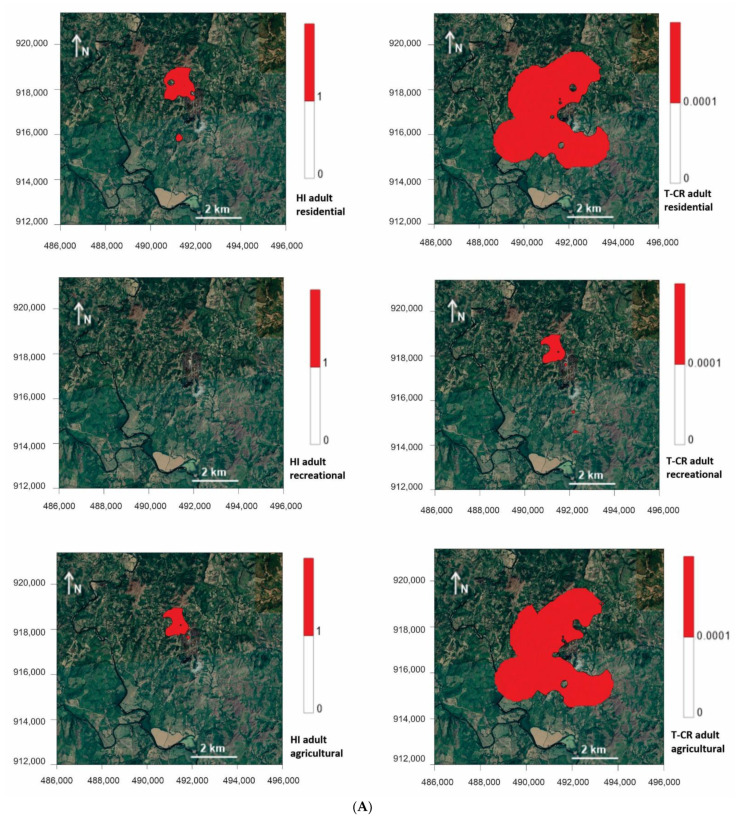

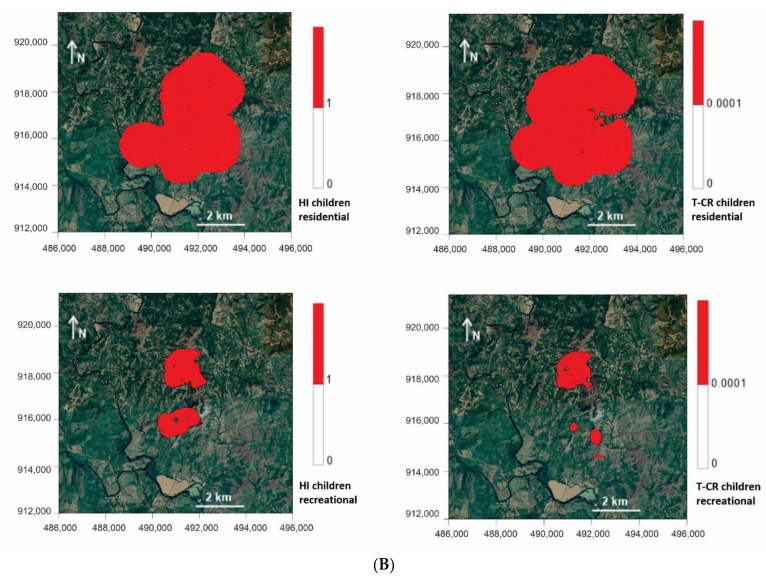
(**A**) Non-carcinogenic risk and carcinogenic risk maps for adults in the residential, recreational and agricultural scenarios. Risk calculated by the concentration values for soils from surrounding areas, soils from mining areas, stream sediments, terrace sediments, tailings, cyanidation ponds and pithead sediments. (**B**) Non-carcinogenic risk and carcinogenic risk maps for children in the residential and recreational scenarios. Risk calculated by the concentration values for soils from surrounding areas, soils from mining areas, stream sediments, terrace sediments, tailings, cyanidation ponds and pithead sediments.

The limit for HI and HQ risks is 1; those that exceed this value are considered high risk [48]. The average estimated total HI (soil ingestion, dermal contact and inhalation) risks assumed for children and adults in the different scenarios (residential, recreational, agricultural) are presented in Supplementary Table S3. Figure 7B represents the distribution of the HI risks for children in the residential and recreational scenarios. In the residential scenario for children, the value was exceeded in all areas and in this order: pithead sediments > tailings > terrace sediments > stream sediments > soils from the mining area > soils from the surrounding areas> cyanidation ponds (150.31 > 19.03 > 5.29 > 5.12 > 2.18 > 1.67 > 1.30, respectively). In the recreational scenario, the HI limit was exceeded in the following areas: pithead sediments > tailings > terrace sediments > stream sediments (39.53 > 5.00 > 1.39 > 1.35, respectively) (Table S3).

The HI map for adults in the residential, agricultural and recreational scenarios is shown in Figure 7A, and the average values are summarized in Supplementary Table S3. The HI risks for adults were surpassed in pithead sediments in both the residential and agricultural scenarios (17.44 and 11.94, respectively), and also in tailings in the same two scenarios (2.20 and 1.51, respectively). This threshold in the recreational scenario was surpassed only in pithead sediments (4.59).

The HI risks were much higher for children than for adults, and the ingestion route contributed the most. In it, As showed the highest values (exceeding HQ). The HQ value through which HI risks were represented by PTEs was evaluated and exceeded by As through the ingestion route for children (residential) and adults (residential and agricultural), and through dermal contact for children in the residential scenario.

#### 3.8.2. Carcinogenic Risk (CR)

The acceptable CR risk level was set to equal 1 × 10^−6^ for an individual PTE and to equal 1 × 10^−4^ for the sum of carcinogenic PTEs [19,48]. Values exceeding these are considered CR. The average values of total CR (soil ingestion, dermal contact and inhalation) represented by the materials studied for children and adults in the different scenarios (residential, recreational, agricultural) are presented in Supplementary Table S4. Figure 7B also presents the CR maps for children in the residential and recreational scenarios. For the children in the residential scenario, the following areas were above the acceptable limit: pithead sediments > tailings > stream sediments = terrace sediments > soils from the surrounding areas > soils from the mining area (6.6 × 10^−3^ > 8.6 × 10^−4^ > 3.1 × 10^−4^ = 3.1 × 10^−4^ > 2.3 × 10^−4^ > 1.9 × 10^−4^, respectively). In the recreational scenario, pithead sediments (1.7 × 10^−3^) and tailings (2.3 × 10^−4^) were above the acceptable value.

Figure 7A depicts the CR maps for adults in the residential, agricultural and recreational scenarios; values are presented in Supplementary Table S4. The acceptable value for the residential scenario was exceeded in the following areas: pithead sediments > tailings > stream sediments > terrace sediments > soils from the surrounding areas > soils from the mining area (3.8 × 10^−3^ > 4.9 × 10^−4^ > 1.8 × 10^−4^ > 1.7 × 10^−4^ > 1.3 × 10^−4^ > 1.0 × 10^−4^, respectively). That for the agricultural scenario was exceeded in this order: pithead sediments > tailings > stream sediments = terrace sediments> soils from the surrounding areas (3.5 × 10^−3^ > 4.5 × 10^−4^ > 1.6 × 10^−4^ = 1.6 × 10^−4^ > 1.1 × 10^−4^, respectively). In the recreational scenario, this value was exceeded in pithead sediments (1.0 × 10^−3^) and tailings (1.3 × 10^−4^). CR risks were much higher for children than adults, and the highest risk route was soil ingestion, followed by dermal contact. The acceptable value for a single PTE (1 × 10^−6^) was exceeded for Cu and As in the residential and agricultural scenarios.

## 4. Discussion

Observing the dispersion maps of Remance PTEs (Figure 2) with respect to local legislation, the total concentrations of As and Cu are those that represent a potential impact on the area, taking into consideration that a study of mobile concentrations (bioavailability) is required to ensure that these elements are indeed present in concerning concentrations. The PCA showed that DHA was favored by the presence of OM and cyanide species in soils, terraces and stream sediments. Furthermore, As and Hg were positive factors (PC1) if DHA was present, as Campos et al. (2018) [36] described in a mining and metallurgical complex of Hg. In the Remance mine scenario, only the higher Cu concentrations seemed to affect soil health in terms of DHA levels. This is surprising evidence for a gold mine with cyanidation during the metallurgical process, but it evidences the need to merge some risk indices for an appropriate initial risk assessment. It is worth mentioning that a complete risk assessment involves acquiring a big dataset that includes data on the pollutants in all environmental compartments and local food supplies, as well as a complete study of the local population’s life habits, such as the origin of the food they eat, the time they spend in local polluted areas, among many more [57,58,59].

A first risk assessment stage should involve the precise delimitation of affected areas. For this purpose, it is necessary to apply indices capable of comparing the levels of PTEs in polluted areas with background areas. Although some indices properly describe the release of certain elements to the environment, a complex scenario such as the Remance mine requires combining a group of elements with similar dispersion patterns. As shown in Figure 5, the PLI shows the impact mainly of the tailing area, pithead sediments, and its surroundings, showing how the contamination has spread from the tailings and pithead sediments; the other zone with a lower degree of impact is to the south of the mine, where excavation work took place. The information provided by the PLI data seems more meaningful than the application of single enrichment factors or applying similar indices to single elements [60].

A second stage should provide information about the degree of effects to biota. It is necessary to state that establishing the bioavailability and transfer rates of PTEs can be challenging, but some indices can provide an initial view of these effects to biota based on a generic dataset. Tailings and pithead are the source of contamination by PTEs affecting the surrounding soils and downstream sediments, as evidenced by the Er and RI. Distribution maps delimit two small areas with considerable damage, including mining materials and some sediments downstream. With this simple approach, it is possible to better delimit the areas indicated by PLI that probably affect biota. The final stage must evaluate the effects of these restricted areas on the local population’s health. As some elements of the Remance mine are carcinogenic, a combination of HI and CR indices is needed to better delimit risk areas. As expected, risk areas are small dimensions for children’s recreational use (both HI and CR), but delimit an area with larger CR dimensions for agricultural use. Obviously, HI and CR for children in residential areas appear with larger values, except HI for adults. The main factors related to the distribution of risk areas were the presence of As, Sb and Hg, which is consistent with many other case studies of mining and industrial pollution in China [61]. Another remarkable finding was that the CR risks for adults and for agricultural uses were wider than expected, as the RI only revealed mild damage for soil samples. Looking at the pollutants that exceeded the accepted levels of risk to human health, the main one was As because it can cause skin, liver and lung cancer, while excess Cu can cause abnormalities of the nervous system [17].

The ecological and health risk assessments of the Remance area revealed that these mining areas are complex scenarios in which many (synergistic and antagonistic) factors must be considered. Although soil health did not seem to be affected by the presence of PTEs, or was even positively affected by them, these PTEs can pose a high risk in some areas, especially areas with recreational uses for children and agricultural uses.

## 5. Conclusions

The abandoned Remance mine poses a risk for the health of the environment and its inhabitants, as different indicators revealed. In particular, the RI best expressed the environmental risk, which coincided with the results of CR and HI to human health.

The areas with the highest risk were pithead sediments, tailings, terrace sediments and stream sediments, which were the areas that still had a strong impact due to mining activity and, to a lesser degree, impacted the mining work area, cyanidation ponds and soils. These results were corroborated by soil health as assessed through DHA, which showed that soil health was better than that of terrace sediments and stream sediments.

According to Er and PI, the elements that posed a risk for the environment were As > Hg > Sb > Cu. The main HI was constituted by As through ingestion and dermal contact, and As and Cu for the CR through ingestion and dermal contact. In all cases, the worst scenario was for children, and for adults in residential, recreational and agricultural scenarios. This is a major concern because peasants live in the study area with their families, and they perform agricultural work and live there on a generally permanent basis.

From a human health point of view, the most relevant pollutant was As because it can cause skin, liver and lung cancer and represented HI and CR through ingestion and dermal contact pathways. More details on the bioavailability of elements are needed in order to realistically assess the risks related to the presence of this element in the area.

Given all of the above considerations for both environmental and human health, it is necessary to set up environmental management programs in these areas to establish the best remediation strategies [62,63,64] and help preserve the right to health and to live in a clean environment.

## Figures and Tables

**Table 1:** Evaluation criteria for the pollution index (PI), the pollution load index (PLI), the toxicity response coefficient (Er) and the potential ecological risk index (RI).

Index	No Polluted	Slightly Polluted	Moderately Polluted	Considerably Polluted	Seriously Polluted	Extremely Polluted
PLI/PI	<1	1 < PLI < 2	2 < PLI < 3	PLI > 3	-	-
Er	<10	<40	40 < Er < 80	80 < Er < 160	160 < Er < 320	Er > 320
RI	<50	<150	150 < RI < 300	300 < RI < 600	600 < RI < 1200	RI > 1200

**Table 2:** The exposure parameters used for the study calculations.

Parameter	Unit	Residential		Recreational		Agricultural
		Adult	Child	Adult	Child	Adult
Exposure frequency (EF)	day year^−1^	365	365	96	96	250
Exposure duration (ED)	year	30 ^a^	6	30 ^a^	6	40
Averaging time (AT)	year day					
Non carcinogenic		ED × EF	ED × EF	ED × EF	ED × EF	ED × EF
Carcinogenic		70 × EF	70 × EF	70 × EF	70 × EF	70 × EF
Ingestion						
Ingestion rate in soil (IR_ing_)	mg kg^−1^	100	200	100	200	100
Fraction ingested (FI)	unitless	1	1	1	1	1
Dermal contact						
Adherence factor (AF)	mg (cm^2^ event)^−1^	0.07	0.2	0.07	0.2	0.07
Event frequency (EV)	events day^−1^	1	1	1	1	1
Skin surface area contact (SA)	cm^2^	6032	2373	6032	2373	6032 ^b^
Inhalation						
Inhalation rate (IR_air_)^a^	m^3^ day^−1^	20	10	20	10	20
Particulate emission factor (PEF)	m^3^ kg^−1^	1.36 × 10^9^	1.36 × 10^9^	1.36 × 10^9^	1.36 × 10^9^	1.36 × 10^9^

^a^ Values taken from Department of Environmental Affairs [47]. ^b^ Value equaling exposure of an adult who engages in agricultural work in the same rural environment. The other values were taken from Gruszecka-Kosowska et al. (2020) [19].

**Table 3:** The toxicological parameters used for the calculations in this study. Abbreviations: RBA, relative bioavailability factor; ABSd, dermal absorption factor; GIABS, fraction of contaminant absorbed in the gastrointestinal tract; RfDo, oral reference doses; RfDd, dermal reference doses; RfDinh, inhalation reference doses; SFo, slope factor for oral; SFd, slope factor for dermal; SFinh, slope factor for inhalation. RBA, ABS and GIABS are unitless, Reference doses (RfD) in mg kg^−1^ day^−1^ and slope factors (SF) in (mg kg^−1^ day^−1^)^−1^.

Element	(Unitless)			RfD (mg kg^−1^ day^−1^)			SF (mg kg^−1^ day^−1^)^−1^		
	RBA ^a^	ABSd ^a^	GIABS ^b^	RfDo ^b^	RfDd ^c^	RfDinh ^d^	Sfo ^e^	SFd ^c^	Sfinh ^d^
Cu	1	0.01	1	4.0 × 10^−2^	4.0 × 10^−2^	-	1.7	1.7	-
Zn	1	0.01	1	3.0 × 10^−1^	3.0 × 10^−1^	-	-	-	-
As	0.6	0.03	1	3.0 × 10^−4^	3.0 × 10^−4^	3.0 × 10^−4^	1.5	1.5	15
Sb	1	0.01	0.15	4.0 × 10^−4^	2.7 × 10^−3^	-	-	-	-
Ba	1	0.01	0.07	2.0 × 10^−1^	2.9 × 10^0^	-	-	-	-
Hg	1	0.01	0.07	3.0 × 10^−4^	4.3 × 10^−3^	8.6 × 10^−5^	-	-	-

^a^ Values taken from Gruszecka-Kosowska et al. (2020) [17]. ^b^ Values taken from USEPA [50]. ^c^ Values calculated according to Wcislo et al. (2016) [49] and Gruszecka-Kosowska et al. (2020) [19]. ^d^ Values taken from Kamunda et al. (2016) [1]. ^e^ Values taken from Pan et al. (2019) [5].

**Table 4:** Average values and standard deviation of the physicochemical parameters and PTEs (mg kg^−1^) in the different material types in the sampled areas.

Sample Type	N	pH	EC	OM (%)	Cu	Zn	As	Sb	Ba	Hg
			(dS m^−1^)							
Soils (mining area) ^a^	19	5.9 ± 0.5	0.03 ± 0.03	8.2 ± 3.8	61.3 ± 51.8	46.9 ± 23.9	56.4 ± 54.4	13.7 ± 2.6	200.6 ± 123.5	0.11 ± 0.16
Soils (surrounding areas) ^a^	24	5.2 ± 0.4	0.05 ± 0.04	5.1 ± 2.0	93.5 ± 85.6	62.8 ± 35.9	35.5 ± 44.2	16.4 ± 5.4	349.7 ± 198.0	0.06 ± 0.06
Terrace sediments ^a^	20	5.3 ± 1.0	0.14 ± 0.22	4.3 ± 2.2	65.2 ± 32.7	55.3 ± 18.8	152.7 ± 210.3	19.8 ± 8.9	355.7 ± 115.0	0.62 ± 1.48
Stream sediments ^a^	19	5.8 ± 0.5	0.07 ± 0.08	3.4 ± 2.7	74.5 ± 40.0	66.3 ± 34.1	143.4 ± 131.7	23.2 ± 12.5	398.7 ± 125.5	0.21 ± 0.25
Tailings ^b^	5	4.1 ± 0.8	0.29 ± 0.57	0.8 ± 0.2	77.7 ± 107.4	27.0 ± 15.2	614.1 ± 222.7	17.8 ± 5.6	376.2 ± 164.6	1.37 ± 1.12
Cyanidation ponds ^b^	4	4.9 ± 0.2	0.09 ± 0.02	5.3 ± 1.7	10.9 ± 7.0	37.1 ± 22.9	41.0 ± 19.7	1.4 ± 0.7	253.9 ± 64.8	0.17 ± 0.07
Pithead sediments ^b^	1	3.9	0.52	12.9	403.0	153.0	5030.0	2.2	55.4	0.62

^a^ This work. ^b^ Values according to González-Valoys et al. (2021) [26].

**Table 5:** Soil guidelines for the PTEs for Panama and Costa Rica (all values are expressed as mg kg^−1^).

Soil Guidelines	Uses	Cu	Zn	As	Sb	Ba	Hg	Reference
Panama Maximum permissible limits of soil contaminants for human health	Others		3	4		10	1.4	[51]
	Residential		30	20		100	14	
	Industrial		300	30		1000	140	
Costa Rica	Prevention Value	20	300	5	2	150	0.5	[52]
Intervention value (Concentration above which there are potential direct or indirect risks to human health)	Agricultural	20	450	35	5	300	12	
	Residential	50	1000	55	10	500	36	
	Industrial	100	2000	150	25	750	70	

**Table 6:** The estimated percent abundance of the mineral phases identified for the selected samples.

Sample	Sample Type	Kaolinite (%)	Illite (%)	Chlorite (%)	Quartz (%)	Feldspar (%)
TR EU 3	Terrace sediments	traces	n.d.	n.d.	100	n.d.
TR J1	Terrace sediments	10	n.d.	traces	85	5
TR J2	Terrace sediments	5	n.d.	n.d.	90	5
TR J3	Terrace sediments	15	5	n.d.	75	5
TR R2	Terrace sediments	15	n.d.	n.d.	85	n.d.
TR R9	Terrace sediments	15	n.d.	n.d.	85	n.d.
TRB	Terrace sediments	15	n.d.	5	80	n.d.
TERR 17	Terrace sediments	10	n.d.	5	75	10
SDEU2	Stream sediments	10	n.d.	25	65	n.d.
SDJ2	Stream sediments	10	n.d.	n.d.	90	n.d.
SDJ3	Stream sediments	10	n.d.	5	85	n.d.
SDR2	Stream sediments	15	n.d.	n.d.	85	n.d.
SDR9	Stream sediments	15	n.d.	n.d.	85	n.d.
SED17	Stream sediments	5	n.d.	traces	95	n.d.
SA6S	Soils (surrounding areas)	10	n.d.	n.d.	90	n.d.
SR5S	Soils (surrounding areas)	10	n.d.	n.d.	90	n.d.
SR6S	Soils (surrounding areas)	10	n.d.	n.d.	90	n.d.
S2LS	Soils (surrounding areas)	10	5	n.d.	85	n.d.

n.d.: not detected; traces: signal appears but is below the detection limit (<100 ppm).

**Table 7:** Summary of the average values and standard deviation of edaphic parameters, DHA (in µg TPF g^−1^ d^−1^) and concentrations of PTEs and cyanide expressed in mg kg^−1^. Abbreviations: EC, electrical conductivity; ORP, oxidation reduction potential; OM, organic matter; CEC, cationic exchange capacity; T-CN, total cyanide; C-CN, complex cyanide; and E-CN, easily released cyanide.

Sample Type	pH	EC dS m^−1^	ORP mV	OM %	CEC cmol kg^−1^	Cu	Zn	As	Sb	Ba	Hg	DHA	T-CN	C-CN	E-CN
Terrace sediments	5.3 ± 1.0	0.14 ± 0.22	489.2 ± 132.6	4.2 ± 2.2	9.7 ± 2.4	66.0 ± 32.0	56.5 ± 19.1	146.5 ± 206.6	18.8 ± 9.7	365.2 ± 119.8	0.59 ± 1.44	37.47 ± 43.31	<1.0	<1.0	<1.0
Stream sediments	5.7 ± 0.6	0.07 ± 0.05	441.3 ± 117.3	3.4 ± 2.4	9.1 ± 2.0	74.0 ± 59.0	77.7 ± 44.3	188.9 ± 145.3	25.5 ± 13.0	429.5 ± 137.8	0.30 ± 0.31	24.79 ± 34.13	1.3 ± 1.5	1.2 ± 1.1	<1.0
Soils (surrounding areas)	5.3 ± 0.4	0.07 ± 0.05	507.0 ± 86.0	5.3 ± 2.2	10.6 ± 4.5	111.7 ± 127.4	80.4 ± 45.6	55.7 ± 50.7	19.0 ± 6.0	430.8 ± 127.1	0.08 ± 0.08	63.27 ± 43.39	1.0 ± 0.8	<1.0	<1.0

**Table 8:** Principal component analysis matrix for the relationship between DHA and edaphic parameters. Numbers in bold correspond to PC1 or PC2 more significative.

Variable	PC1	PC2
pH	−0.131	0.319
EC	0.034	−0.141
ORP	**−0.334**	−0.204
OM	**0.379**	0.247
CEC	**0.202**	0.177
Cu	−0.018	−0.019
Zn	0.074	0.048
As	0.141	**−0.489**
Sb	0.044	**−0.424**
Ba	**−0.160**	−0.161
Hg	0.154	**−0.423**
DHA	**0.384**	0.190
T-CN	**0.464**	−0.125
C-CN	**0.369**	−0.227
E-CN	**0.333**	0.122

**Table 9:** The PI and PLI average values and standard deviation calculated for the different sample types in the Remance mine area.

Type	PI Cu	PI Zn	PI As	PI Sb	PI Ba	PI Hg	PLI
Soils (mining area) ^a^	1.3 ± 1.1	0.6 ± 0.3	1.4 ± 1.4	0.9 ± 0.2	0.4 ± 0.2	1.6 ± 2.4	0.7 ± 0.3
Soils (surrounding areas) ^a^	1.9 ± 1.8	0.8 ± 0.5	0.9 ± 1.1	1.1 ± 0.4	0.7 ± 0.4	1.0 ± 0.9	0.7 ± 0.4
Terrace sediments ^a^	0.8 ± 0.4	0.7 ± 0.2	5.4 ± 7.4	6.6 ± 3.0	0.7 ± 0.2	15.9 ± 37.9	1.9 ± 1.2
Stream sediments ^a^	1.1 ± 0.6	0.9 ± 0.5	5.8 ± 5.3	1.3 ± 0.7	0.8 ± 0.2	6.1 ± 7.5	1.6 ± 0.8
Tailings ^b^	11.1 ± 15.3	1.0 ± 0.5	33.0 ± 12.0	59.3 ± 18.7	1.6 ± 0.7	9.1 ± 7.5	7.3± 3.0
Cyanidation ponds ^b^	1.6 ± 1.0	1.3 ± 0.8	2.2 ± 1.1	4.7 ± 2.4	1.1 ± 0.3	1.1 ± 0.5	1.6 ± 0.3
Pithead sediments ^b^	57.6	5.5	270.4	7.3	0.2	4.1	9.2

^a^ This work. ^b^ Values calculated with the data obtained from González-Valoys et al., 2021 [26].

**Table 10:** Average values and standard deviation of Er and RI for the different sample types.

Type	Er Cu	Er Zn	Er As	Er Sb	Er Hg	RI
Soils (surrounding area)	9.5 ± 8.8	0.8 ± 0.5	8.9 ± 10.8	7.8 ± 2.5	38.1 ± 35.4	65 ± 44
Soils (mining area)	6.4 ± 5.4	0.6 ± 0.3	14.1 ± 13.6	6.5 ± 1.2	65.5 ± 97.4	93 ± 108
Terrace sediments	4.1 ± 2.0	0.7 ± 0.2	51.6 ± 72.7	44.2 ± 22.1	605.6 ± 1481.2	706 ± 1554
Stream sediments	5.5 ± 2.9	0.9 ± 0.5	55.3 ± 52.8	9.0 ± 4.8	233.1 ± 295.0	304 ± 337
Tailings ^a^	55.5 ± 76.7	1.0 ± 0.5	330.2 ± 119.7	415.1 ± 131.1	365.9 ± 299.0	1168 ± 509
Cyanidation ponds ^a^	7.8 ± 5.0	1.3 ± 0.8	22.0 ± 10.6	32.7 ± 16.9	44.7 ± 19.6	109 ± 32
Pithead sediments ^a^	287.9	5.5	2704.3	51.3	165.3	3214

^a^ Values calculated with the data obtained from González-Valoys et al., 2021 [26].

## Data Availability

Not applicable.

## Supplementary Materials

The following are available online at https://www.mdpi.com/article/10.3390/ijerph18179369/s1. Table S1: Sample codes and typology, along with geographic coordinates, Table S2: Result of samples, Table S3: Non-carcinogenic risk, Table S4: Carcinogenic risk, Figure S1: Diffractograms.

## References

[1] Kamunda C., Mathuthu M., Madhuku M. (2016). Health Risk Assessment of Heavy Metals in Soils from Witwatersrand Gold Mining Basin, South Africa. Int. J. Environ. Res. Public Health.

[2] Ramappa H., Muniswamy D. (2018). Spatial Distribution of Heavy Metals around the Gold Mine Ore Tailings of Hatti, Karnataka State, India. Landsc. Environ..

[3] Kaninga B., Chishala B., Maseka K., Sakala G., Lark M., Tye A., Watts M. (2020). Review: Mine tailings in an African tropical environment-mechanisms for the bioavailability of heavy metals in soils. Environ. Geochem. Health.

[4] Elmayel I., Esbrí J., García-Ordiales E., Elouaer Z., García-Noguero E., Bouzid J., Campos J., Higueras P. (2020). Biogeochemical assessment of the impact of Zn mining activity in the area of the Jebal Trozza mine, Central Tunisia. Environ. Geochem. Health.

[5] Pan Y., Peng H., Xie S., Zeng M., Huang C. (2019). Eight Elements in Soils from a Typical Light Industrial City, China: Spatial Distribution, Ecological Assessment, and the Source Apportionment. Int. J. Environ. Res. Public Health.

[6] Sadras V., Alston J., Aphalo P., Connor D., Denison R.F., Fischer T., Gray R., Hayman P., Kirkegaard J., Kirchmann H. (2020). Chapter Four-Making science more effective for agriculture. Adv. Agron..

[7] Silveira M., Kohmann M. (2020). Chapter 3—Maintaining soil fertility and health for sustainable pastures. Management Strategies for Sustainable Cattle Production in Southern Pastures.

[8] Kumar S., Chaudhuri S., Maiti S. (2013). Soil dehydrogenase enzyme activity in natural and mine soil—A review. Middle-East J. Sci. Res..

[9] Wolinska A., Stepniewska Z. (2012). Dehydrogenase Activity in the Soil Environment. Dehydrogenase.

[10] Zhang N., He X., Gao Y., Li Y., Wang H., Ma D., Zhang R., Yang S. (2010). Pedogenic Carbonate and Soil Dehydrogenase Activity in Response to Soil Organic Matter in Artemisia ordosica Community. Pedosphere.

[11] Gallego S., Esbrí J.M., Campos J.A., Peco J.D., Martin-Laurent F., Higueras P. (2021). Microbial diversity and activity assessment in a 100-year-old lead mine. J. Hazard. Mater..

[12] Kierczak J., Neel C., Aleksander-Kwaterczak U., Helios-Rybicka E., Bril H., Puziewicz J. (2008). Solid speciation and mobility of potentially toxic elements from natural and contaminated soils: A combined approach. Chemosphere.

[13] Rodríguez-Hernández A., Lázaro I., Razo I., Briones-Gallardo R. (2021). Geochemical and mineralogical characterization of stream sediments impacted by mine wastes containing arsenic, cadmium and lead in North-Central Mexico. J. Geochem. Explor..

[14] Bravo S., Amorós J., Pérez de los Reyes C., García F., Moreno M., Sánchez-Ormeño M., Higueras P. (2015). Influence of the soil pH in the uptake and bioaccumulation of heavy metals (Fe, Zn, Cu, Pb and Mn) and other elements (Ca, K, Al, Sr and Ba) in vine leaves, Castilla-La Mancha (Spain). J. Geochem. Explor..

[15] Sun Z., Chen J. (2018). Risk Assessment of Potentially Toxic Elements (PTEs) Pollution at a Rural Industrial Wasteland in an Abandoned Metallurgy Factory in North China. Int. J. Environ. Res. Public Health.

[16] Agency for Toxic Substances and Disease Registry (ATSDR) The ATSDR 2019 Substance Priority List. https://www.atsdr.cdc.gov/spl/index.html.

[17] Bini C., Wahsha M., Bini C., Bech J. (2014). Potentially Harmful Elements and Human Health. Book PHEs, Environment and Human Health: Potentially Harmful Elements in the Environment and the Impact on Human Health.

[18] Agency for Toxic Substances and Disease Registry (ATSDR) (2006). Toxicological Profile for Cyanide.

[19] Gruszecka-Kosowska A., Baran A., Wdowin M., Mazur-Kajta K., Czech T. (2020). The content of the potentially harmful elements in the arable soils of southern Poland, with the assessment of ecological and health risks: A case study. Environ. Geochem. Health.

[20] Dirección de Hidrometeorología de ETESA Mapa de Clasificación Climática (según Köppen) de Panamá, año 2007. http://www.hidromet.com.pa/mapas.php.

[21] Instituto Geográfico Nacional Tommy Guardia (IGNTG) (1988). Atlas Nacional de la República de Panama.

[22] Gómez A. (2008). Contaminación Ambiental en Áreas Asociadas con Minas Antiguas de oro. Determinación de Cianuro en Agua y de Trazas Metálicas en Sedimentos, en las Quebradas Aledañas a las Minas Remance y Santa Rosa. Master’s Thesis.

[23] Nelson C., Ganoza J. (1999). Mineralización de oro en la franja aurífera de Veraguas, Panamá. Rev. Geol. Am. Cent..

[24] Hughes W. (1998). Minería ¿Desarrollo o Destrucción?.

[25] Radio Temblor Continúan las Protestas Contra la Minería y Corrupción en Panamá (Reactivan Mina de Remance, en Veraguas). https://www.radiotemblor.org/continuan-las-protestas-contra-la-mineria-y-corrupcion-en-panama-reactivan-mina-remance-en-veraguas/.

[26] González-Valoys A., Arrocha J., Monteza-Destro T., Vargas-Lombardo M., Esbrí J., García-Ordiales E., Jiménez-Ballesta R., García-Navarro F., Higueras P. (2021). Environmental challenges related with cyanidation in Central American gold mining, Remance mine (Panama). J. Environ. Manag..

[27] Sánchez-Donoso R., Martín Duque J.F., Crespo Feo E., Higueras P. (2019). Tailing’s geomorphology of the San Quintín mining site (Spain): Landform catalogue, aeolian erosion and environmental implications. Environ. Earth Sci..

[28] Ministerio de Ambiente Panamá (2012). Mapa de Cobertura y Uso de la Tierra, en la República de Panamá del Año.

[29] American Society Testing of Materials (ASTM) (2004). Annual Book of ASTM Standars.

[30] Weaver R., Syers J., Jackson M. (1991). Análisis Químico de Suelos.

[31] Porta J. (1986). Técnicas y Experimentos en Edafología.

[32] Molina J.A., Oyarzun R., Esbrí J.M., Higueras P. (2006). Mercury accumulation in soils and plants in the Almadén mining district, Spain: One of the most contaminated sites on earth. Environ. Geochem. Health.

[33] Internacional Organization for Standardization (ISO) (2012). Water Quality—Determination of Total Cyanide and Free Cyanide Using Flow Analysis (FIA and CFA)—Part 2: Method Using Continuous Flow Analysis (CFA) (ISO 14403-2).

[34] Casida L., Klein D., Santoro T. (1964). Soil Dehydrogenase Activity. Soil Sci..

[35] Tan X., Liu Y., Yan K., Wang Z., Lu G., He Y., He W. (2017). Differences in the response of soil dehydrogenase activity to Cd contamination are determined by the different substrates used for its determination. Chemosphere.

[36] Campos J.A., Esbrí J.M., Madrid M.M., Naharro R., Peco J., García-Noguero E.M., Higueras P. (2018). Does mercury presence in soils promote their microbial activity? the Almadenejos case (Almadén mercury mining district, Spain). Chemosphere.

[37] Rietveld H. (1969). A profile refinement method for nuclear and magnetic structures. J. Appl. Cryst..

[38] Rodríguez-Carvajal J. (1993). Recent advances in magnetic structure determination by neutron powder diffraction. Phys. B.

[39] Young R.A. (1995). The Rietveld Method.

[40] García-Lorenzo M., Crespo-Feo E., Esbrí J., Higueras P., Grau P., Crespo I., Sánchez-Donoso R. (2019). Assessment of potentially toxic elements in technosols by tailings derived from Pb-Zn-Ag mining activities at San Quintín (Ciudad Real, Spain): Some insights into the importance of integral studies to evaluate metal contamination pollution hazards. Minerals.

[41] Tomlinson D., Wilson J., Harris C., Jeffrey D. (1980). Problems in the assessment of heavy-metal levels in estuaries and the formation of a pollution index. Helgoländer Meeresunters.

[42] Hakanson L. (1980). An ecological risk index for aquatic pollution control. A sedimentological approach. Water Res..

[43] Wang N., Wang A., Kong L., He M. (2018). Calculation and application of Sb toxicity coefficient for potential ecological risk assessment. Sci. Total Environ..

[44] U.S. Environmental Protection Agency (2001). Risk Assessment Guidance for Superfund, Vol. 3: Part A, Process for Conducting Probabilistic Risk Assessment.

[45] U.S. Environmental Protection Agency (2004). Risk Assessment Guidance for Superfund, Vol. 1: Human Health Evaluation Manual (Part E, Supplemental Guidance for Dermal Risk Assessment), Final.

[46] U.S. Environmental Protection Agency (2009). Risk Assessment Guidance for Superfund Volume I: Human Health Evaluation Manual (Part F, Supplemental Guidance for Inhalation Risk Assessment) Final.

[47] Department of Environmental Affairs The Framework for the Management of Contaminated Land, South Africa, Year 2010. http://sawic.environment.gov.za/documents/562.pdf.

[48] U.S. Environmental Protection Agency (1989). Risk Assessment Guidance for Superfund, Vol. 1: Human Health Evaluation Manual, Part A, Interim Final.

[49] Wcisło E., Bronder J., Bubak A., Rodriguez-Valdés E., Gallego J.L.R. (2016). Human health risk assessment in restoring safe and productive use of abandoned contaminated sites. Environ. Int..

[50] U.S. Environmental Protection Agency (2019). Regional Screening Level (RSL) Summary Table (TR = 10−6, HQ = 1), April 2019.

[51] Gaceta Oficial Digital de Panamá (2009). Decreto Ejecutivo N° 2 “Por el cual se establece la Norma Ambiental de Calidad de Suelos para diversos usos”.

[52] Ministerio de Salud (2010). Reglamento Sobre Valores Guía en Suelos Para Descontaminación 602 de Sitios Afectados por Emergencias Ambientales y Derrames.

[53] Palansooriyaa K., Shaheenb S., Chene S., Tsange D., Hashimotof Y., Houg D., Bolanh N., Rinklebeb J., Oka Y. (2020). Soil amendments for immobilization of potentially toxic elements in contaminated soils: A critical review. Environ. Int..

[54] Sabadini-Santos E., Castilhos Z.C., Bidone E.D. (2020). Microbial Activities Response to Contamination in Soil and Sediments Rich in As Surrounding an Industrial Gold Mine. Water Air Soil Pollut..

[55] Meier S., Curaqueo G., Khan N., Bolan N., Rilling J., Vidal C., Borie F. (2017). Effects of biochar on copper immobilization and soil microbial communities in a metal-contaminated soil. J. Soils Sediments.

[56] Mukhopadhyay S., Maiti S.K. (2010). Dehydrogenase activity in natural and mine soil—A review. Indian J. Environ. Prot..

[57] Iribarren I., Chacón E., De Miguel E. (2009). A Bayesian approach to probabilistic risk assessment in municipal playgrounds. Arch. Environ. Contam. Toxicol..

[58] Harris M., Stinson J., Landis W. (2017). A Bayesian approach to integrated ecological and human health risk assessment for the South river, Virginia mercury-contaminated site. Risk Anal..

[59] Jiménez-Oyola S., García-Martínez M., Ortega M.F., Bolonio D., Rodríguez C., Esbrí J.M., Higueras P. (2020). Multi-pathway human exposure risk assessment using Bayesian modeling at the historically largest mercury mining district. Ecotoxicol. Environ. Saf..

[60] Hossen M.A., Chowdhury A.I.H., Mullick M.R.A., Hoque A. (2021). Heavy metal pollution status and health risk assessment vicinity to Barapukuria coal mine area of Bangladesh. Environ. Nanotechnol. Monit. Manag..

[61] Chen X., Li F., Zhang J., Liu S., Ou C., Yan J., Sun T. (2021). Status, fuzzy integrated risk assessment, and hierarchical risk management of soil heavy metals across China: A systematic review. Sci. Total Environ..

[62] Liang J., Feng C., Zeng G., Gao X., Zhong M., Li X., Li X., He X., Fang Y. (2017). Spatial distribution and source identification of heavy metals in surface soils in a typical coal mine city, Lianyuan, China. Environ. Pollut..

[63] Hosseini M., Rezazadeh M., Salimi A., Ghorbanli M. (2018). Distribution of heavy metals and arsenic in soils and indigenous plants near an iron ore mine in northwest Iran. Acta Ecol. Sin..

[64] Sun Z., Xie X., Wang P., Hu Y., Cheng H. (2018). Heavy metal pollution caused by small-scale metal ore mining activities: A case study from a polymetallic mine in South China. Sci. Total Environ..

