# Supplementary figures and images for: Ecological and Health Risk Assessments of an Abandoned Gold Mine (Remance, Panama): Complex Scenarios Need a Combination of Indices
Source: Int J Environ Res Public Health. 2021 Sep 5;18(17):9369. doi: 10.3390/ijerph18179369 (PMC8431601; doi:10.3390/ijerph18179369)

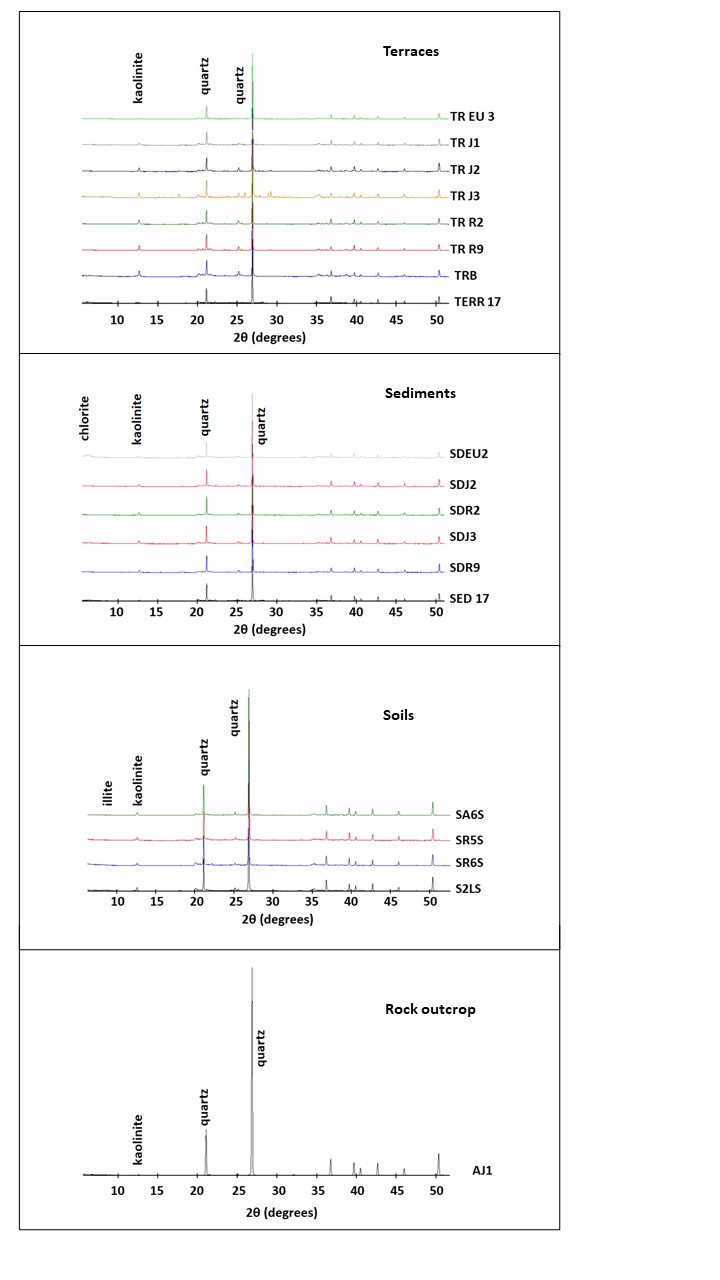

Supplement: Supplementary file 1 [file ijerph-18-09369-s001.zip › Suplementary figure-S1.jpg]
